# Using intervention mapping to develop an intervention for multiparty communication with people with congenital deafblindness

**DOI:** 10.1371/journal.pone.0299428

**Published:** 2024-05-09

**Authors:** Mijkje Worm, Saskia Damen, Marleen J. Janssen, Alexander E. M. G. Minnaert

**Affiliations:** 1 Bartiméus, Zeist, The Netherlands; 2 Pedagogical and Educational Sciences, Nieuwenhuis Institute, University of Groningen, Groningen, The Netherlands; 3 Royal Kentalis, Kentalis Academy, Utrecht, The Netherlands; Örebro University Faculty of Medicine and Health: Orebro universitet Fakulteten for medicin och halsa, SWEDEN

## Abstract

**Background:**

Due to their dual sensory impairment, people with congenital deafblindness (CDB) are rarely naturally involved in other people’s conversations. Their communication partners find it challenging to include them in group conversations. However, overhearing others communicate is important for developing social and communication skills. Hence, we developed an intervention program to guide communication partners in offering multiparty communication to people with CDB. This article describes how the program was developed through an intervention mapping approach.

**Method:**

Intervention mapping is a six-step process: logic model, model of change, program design, program production, program implementation plan, and evaluation plan. These six steps were applied to systematically develop a program to foster multiparty communication in people with CDB. Representatives of the involved groups participated in the project group and the working group to ensure feasibility and acceptability.

**Results:**

Following the intervention mapping steps resulted in creation of a program for communication partners that consists of an education session, practicals, and four video-feedback sessions. Information sessions for practitioners and managers were also developed. The program was implemented incrementally with program implementers in each organization. A subjective evaluation and an impact evaluation were done after each implementation phase.

**Discussion:**

Intervention mapping was used to develop a program that connects theory to practice. The program appeared to meet the communication partners’ needs and be feasible in terms of time investment. This article offers suggestions for broadening the scope of the program to other settings and for further investigating the effects of the program on the social and communication skills of people with CDB.

## Introduction

People with congenital deafblindness (CDB) have disabilities in both vision and hearing that originate *in utero*, at birth, or shortly after, but before the onset of language development [1]. Due to these sensory disabilities, spoken language is inaccessible to them and they have difficulty developing communication skills. This leads to challenges when learning to communicate about referents that are not present in the here-and-now situation, also known as symbolic communication [2]. Even when symbolic communication is achieved, it is typically based on alternative means of communication, including referential objects, social haptic communication, and tactile and non-tactile gestures, signs, and pictograms. Full linguistic understanding and expression are uncommon for people with CDB. Their dual sensory impairment further implies that proximity to the communication partner(s) is essential to conveying or receiving communicative messages [3].

Since communicating with others is a daily challenge for people with CDB, it has become natural to prioritize communication in support programs and interventions. In fact, some argue that communication with people with CDB can only emerge as a result of intervention [4]. In the last decade, an increasing number of such interventions have proven effective [5]. Typically, these interventions are based on video-feedback coaching. In video-feedback coaching, a coach guides communication partners to evaluate their own behavior in interactions with people with CDB through video recordings of their own interactions [5]. Video-feedback interventions that aim to improve communication with people with CDB focus on several aspects of interaction and communication, like intersubjective communication [6], affective involvement [7], or bodily emotional traces [8].

Generally, these interventions focus on one-on-one (dyadic) communicative settings. Dyadic interactive situations are generally considered to be the optimal situation for the learning and development of people with deafblindness [3], as they allow for intensive mutual attunement. Furthermore, strategies for improving communication with people with deafblindness are usually exemplified by dyadic communication practices [3, 9, 10].

However, dyadic communication places considerable demands on communication partners. In dyadic communication, each communicative expression is directly addressed to the other and typically requires a reaction. Individual participants are assumed to experience less pressure to respond when interacting with multiple people simultaneously, since this situation allows them to wait for others to respond [11]. Although coordinating between two others can be more challenging than dyadic communication, infants as young as 3 months old are able to handle these conversations with adults [12]. Researchers have illustrated that by the age of 6–9 months, children can even coordinate socially in a group of three peers [13].

Conversations between three or more people, called multiparty communication (MPC), offer great potential for developing social skills among small children. The presence of a third person increases the number of interactive contexts. For example, this conversation type provides greater opportunities for differentiating the self from others [12]. Also, some authors considered it easier to participate in a conversation with an ongoing topic than to initiate a new topic [11]. This was attributed to a scaffolding effect that comes from knowing the communication partners’ interests. Lastly, scholars have highlighted the potential of a multiparty communicative setting for learning how social relationships are established and maintained [12, 14]. These authors state that even when children are not actively engaged in a conversation but merely listen in with a participant-observer perspective, they learn how affect is shared between individuals [12].

Observing the behavior of others is an important resource for making daily decisions [15]. Modeled behavior and its consequences present different aspects of language and cultural membership than direct dyadic communication [16]. Indeed, humans often watch other people’s behavior and observe its consequences. This observational behavior—known as vicarious capability—is a means of learning about interaction with the physical environment and social interaction [15]. In particular, witnessing communication between others demonstrates aspects of sociability and increases one’s understanding of relationships [12, 14]. Furthermore, children learn new words by overhearing other people’s communication [17].

For a person with CDB, casual observation of dyadic communication between others is unlikely; in order to observe this communication, they need to be close enough, usually even in physical contact. Therefore, they can only observe communication between others in conversation types where more than two people are involved, like triadic of tetradic communication. The general term for these conversation types involving more than two people is multiparty communication [18]. In contrast to verbal communication between seeing-hearing communication partners, natural interactions with people with CDB barely include MPC [19].

Lundqvist [18] raised this issue a decade ago, leading to a multitude of case reports in contributions at Deafblind International World Conferences and European Conferences over the past decade [20]. Presentations and workshops at these conferences reported the effects of introducing MPC to one or a few people with CDB. Although they present successes in terms of personal and social development, the application of MPC usually requires effort and dedication from the communication partners.

Current interventions to improve communication and social interaction for people with CDB are, by default, based on dyadic instructive situations. However, dyadic communication offers relatively fewer opportunities for vicarious learning than MPC. Although some people with CDB have been successfully introduced to MPC, it required time and effort from their communication partners and it did not naturally transfer to interactions with other people with CDB. According to some authors, MPC is especially challenging in the tactile modality [19].

The aim of this research project was to develop an MPC program that supports professional caregivers in fostering MPC in people with CDB. The intervention mapping approach was applied to the development of this program. Intervention mapping [21] is a method of developing interventions with a foundation in both theory- and practice-based experiences. It was designed as a protocol for developing health promotion programs, and it has proven to be effective in many programs designed to enhance health-related behavior (for an overview, see [21]). Recent intervention mapping programs include a self-management program for older people [22] and a program for reducing sexual prejudice in schools [23].

The current paper reports on the process of intervention mapping that led to the protocol that guides communication partners toward developing the knowledge, skills, and confidence to offer MPC to people with CDB. In the methods section, we describe the intervention mapping steps and how these were applied. In the results section, we explain how the steps in the intervention mapping process resulted in an MPC intervention, an implementation plan, and an evaluation plan. The paper aims to exemplify how intervention mapping can be applied to develop evidence-based interventions for CDB care. The description of the program for MPC involving people with CDB and the results of the pilot study will be presented in another paper.

## Methods

This study was conducted between January 2021 and March 2023. It was approved by the Ethics Committee for BSS (Behavioral and Social Sciences) at the University of Groningen.

### Intervention mapping

Intervention mapping is a six-step process: 1) logic model of the problem, 2) program outcomes and objectives, 3) program design, 4) program production, 5) program implementation plan, and 6) evaluation plan [21]. The methods and results sections of this article are organized following these intervention mapping steps. However, intervention mapping is not as linear as the stepwise formulation might suggest. It is an iterative, recursive process in which subsequent findings may lead to a reassessment of earlier steps. This high level of flexibility is part of the intervention mapping process [21]. For the purpose of transparency, the process developers recommend systematic reporting on the steps taken. A clear description of both the program and the intervention protocol enables replication, dissemination, and meta-analyses [24].

Intervention mapping is rooted in a social-ecological model, meaning that the focus of the program is on the interrelationships between individuals and their environments. Those environments consist of multiple influencers across domains, such as personal relationships, community influences, and societal influences. To design an effective program, developers need to take these different domains into account [21]. Representatives of the target population and representatives of their multiple environments should participate in the intervention mapping project groups to ensure that their viewpoints are included in the development of the intervention.

### Project groups

Intervention mapping is organized in planning groups and working groups whose participants may change during the process [21]. Table 1 presents an overview of the project groups.

**Table 1:** Project groups.

Group	Members & disciplines	Task
**Planning group**	2 members: researchers/ psychologists	Coordination of the MPC project
**Working group**	9 members: researchers, psychologists, speech therapist, care specialist, teacher, caregivers, manager	Take the intervention mapping steps: complete the needs assessment; develop, implement, and evaluate the MPC program
**International sounding board group**	16 members: researchers and practitioners (EU and US)	Provide feedback on the rationale for the program and the training; share cultural differences; discuss international dissemination
**Research group**	4 members: researchers/psychologists	Develop the scientific foundation of the program and the intervention study

The planning group consisted of the first two authors of this article; they work as researchers and psychologists, and they led the MPC project. They met approximately every eight weeks to discuss how the research was progressing, the theoretical and practical foundation of the intervention, and the issues to be resolved in the working group. The other two authors were involved as supervisors and sounding boards and were particularly involved in the research design.

The working group focused primarily on the content of the program and the implementation of the study. Initially, the group consisted of the first two authors of this article, plus a psychologist, a speech therapist, and a care specialist. The latter three people represented three organizations in the Netherlands that provide services for people with CDB. The working group was expanded during the needs assessment to include a teacher, two caregivers, and a manager. This ensured that these perspectives were actually considered during the development of the program. The working group met five times to complete the first four intervention mapping steps before the pilot study. After the pilot study, a final meeting was organized to evaluate and refine the first steps and to complete the last two steps of the protocol. Working group meetings had a duration of 1.5 to 2 hours.

The working group meetings had a set structure. To open the discussion, the first author presented the results of the steps taken so far. Then the first author led open discussions in which the working group members offered feedback on the program and the underlying materials. The first author concluded these discussions by summarizing the issues discussed, and the participants could respond one last time. The planning group integrated the results of the working group’s discussions into the program.

In addition, we established an international sounding board group to gather experiences with MPC from professionals from various countries. The sounding board group consisted of representatives of people with CDB, researchers, communication partners, and practitioners from Europe and North America. Practitioners are professionals who advise communication partners and are involved in treating people with CDB (e.g., psychologists, coaches, and speech therapists). They discussed the rationale for the program and the training sessions, cultural differences, and international dissemination.

The project’s activities were supported by the research team (i.e., the authors of this article) to ensure the scientific foundation of the program. Their tasks were to reach agreement on the underlying theories, make decisions about the materials, state the program goals, and report the results.

### Step 1: Logic model of the problem

The first step in intervention mapping is to establish a logic model of the problem, depicted in a relationship between the health problems and their causes. The priority population and the intervention’s context are also described in this step. This study drew upon the experiential knowledge of communication partners as the main source of information. We gathered that knowledge through focus group sessions [19] and the participation of communication partners in the working group.

The three focus group sessions were held at three Dutch organizations that provide care and consultancy for people with CDB. The sessions were attended by professional caregivers (n = 15), practitioners (n = 7), and relatives (n = 2). The main question revolved around the added value of MPC in conversations with people with CDB. The sessions also provided valuable information about the behavior and environmental factors associated with the amount of MPC that people with CDB engage in. The results of this focus group study have been published in another paper [19].

To our knowledge, there are no other scientific papers describing the effects of MPC on the development of communication and social skills by people with CDB. Therefore, the authors manually searched scientific and gray literature for publications involving people with CDB that provided information relevant for producing the logic model of the problem. Those publications studied the social interaction and communication of people with CDB.

Additionally, we searched the literature to explore the development of MPC in young children. With this, we wanted to substantiate the statements made in the focus group sessions about the added value of MPC for development. The following keywords were entered into database (ERIC, PubMed) literature searches: (“infants” OR “toddlers” OR “early childhood”) AND (“multipart*” OR “triad*”). Articles published in a peer-reviewed journal as an empirical study or case description(s) focusing on effects of MPC on the child’s social and/or communicative development were selected. We then excluded articles for which the full text was not available, articles written in a language other than English, and articles that described research on nonhumans. Additional references were identified via a manual search. This process resulted in a total of 22 papers that were included in the logic model of the problem. A flowchart of this search is presented in S1 Fig.

Based on the findings from the focus group sessions and the literature study, the planning group and the research team shared the task of reaching consensus on a logic model of the problem. The final task of this first step, according to intervention mapping [21], was to formulate the program goals: the intended changes in health, quality of life, behavioral factors, and/or environmental factors [21].

### Step 2: Program outcomes and objectives

The second step in intervention mapping focuses on establishing the objectives for change. In the current study, the working group performed four intervention mapping tasks to define the program outcomes and objectives for change. First, they identified the stakeholders essential to achieving the program goals: people with CDB, their communication partners, practitioners, and managers. According to the working group, each of these target groups needed to change their behavior for the MPC program to be successful. The needs assessment in step 1 clarified the measurable behavioral and environmental outcomes.

The second task of the working group was to assign the outcomes to lists of performance objectives per stakeholder group. Then, the working group identified determinants. The intervention mapping process defines determinants as factors within the individual that direct their behavior and can be influenced by interventions. Determinants in intervention mapping are usually expressed in general terms on the levels of cognition and skills [21]. The fourth and final task of this step involved developing a model of change and matrixes of change objectives that describe how the determinants need to change to achieve the performance objectives. The research team then reviewed the efforts of the working group, reflected in the model of change and the matrixes.

### Step 3: Program design

The third step in intervention mapping designs the intervention, by matching the change objects to the theoretical models and practical applications. With this intention, we consulted the tables with methods for changing behavior provided by the intervention mapping handbook [21]. These tables list theory- and evidence-based change methods, arranged by determinants. The determinants for the MPC program (selected in step 2) were matched to appropriate change methods. The change methods then were discussed in the working group and with the research team. Next, the working group converted those change methods into practical applications, considering the implementation context and the environment.

### Step 4: Program production

In the fourth step, the program’s scope and sequence, as well as its components and materials, are described in an intervention plan. The theoretical assumptions collected in step 1, the model of change developed in step 2, and the practical applications compiled in step 3 were all used to develop a coherent program to assist communication partners in enhancing MPC with people with CDB. The intervention mapping process suggests that program materials and protocols be drafted and pretested in this step. The program–an intervention on the level of the communication partners–was produced, tested in a pilot study with two cases, evaluated, and finalized.

### Step 5: Program implementation plan

The focus of the implementation plan is to describe the optimal environmental conditions for using MPC in daily communication with people with CDB. With this in mind, the working group clarified the roles, responsibilities, and support of those involved in the program and identified the people who should adopt and implement the program. To enable evaluation of the program and its effects on social and communication skills, it was implemented as similarly as possible in the various settings in the study. For the overall implementation that took place after the study, separate implementation plans were proposed to account for the differences in structure and roles between the organizations. Nevertheless, implementation was always based on the determinants and change objectives. Representatives of each organization were involved in the implementation process at their organization.

### Step 6: Evaluation plan

The research team, who had experience with evaluating the feasibility and acceptability of interventions, proposed the design to assess the outcome of the MPC program. Process evaluation goals were determined based on the program goals that had been produced in the prior steps. Next, the planning team discussed the proposed evaluation methods and materials of data collection and analysis in the working group, evaluating the feasibility and connection to communication partners’ daily practice.

## Results

### Step 1: Logic model of the problem

**Fig 1:**
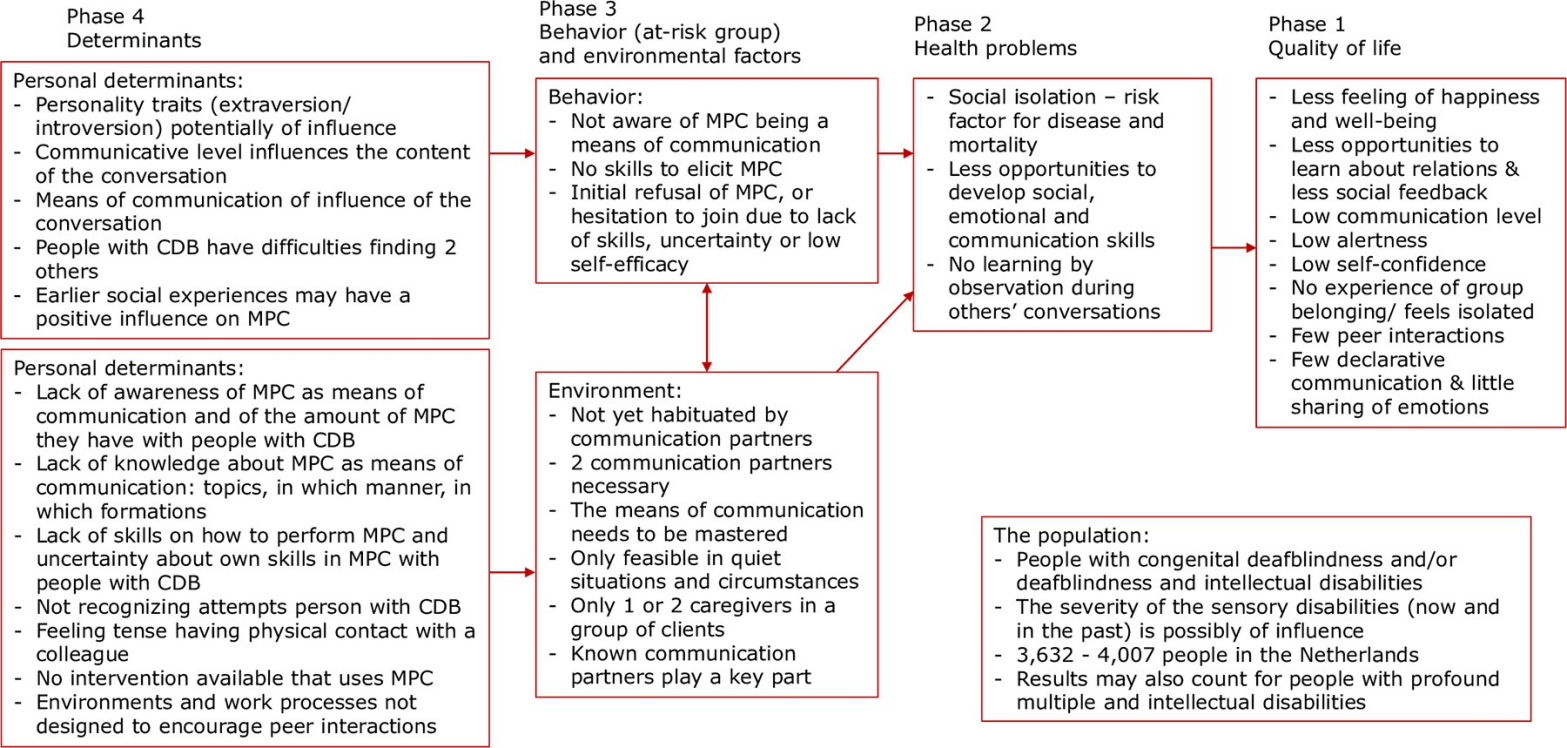
Logic model of the problem.

In this first intervention mapping step, a logic model of the problem was established and the program goals were formulated (Fig 1). The development process for the logic model started by describing the population, the health problem, and the quality-of-life problem. The behavior and environmental factors and determinants were then studied. Once completed, the logic model was considered to be a causal model of the health problem and quality of life. It can be read from left to right, guided by arrows [21].

The logic model for the current study describes the health problems associated with a lack of MPC in relation to behavioral and environmental factors [21] (depicted in Fig 1). The sources for the logic model were the report of the focus group sessions for a preceding study [19], a literature review on people with congenital deafblindness, and a literature review on the development of multiparty communication in the general population. The first author, in close collaboration with the working group, synthesized the results of these sources into the logic model. The program goals were formulated by the working group and validated by the research group.

#### The population

The priority population (i.e., people with CDB) is described in the logic model. An estimated 3,632–4,007 people in the Netherlands have CDB [25]. Common causes of CDB include rubella syndrome and CHARGE syndrome. Other underlying causes are prematurity, infections, birth complications, and other syndromes [26]. The origin of deafblindness in Dutch adults living in residential care is often unclear, as neonatal auditory screening was only introduced nationwide in the 21st century [26, 27]. Hence, although a person’s functioning may suggest a congenital origin of deafblindness, this origin is generally not confirmed in their medical record. All people with CDB need a high level of support in all aspects of everyday life [26].

#### Quality of life & health problem

The next fields in the logic model describe the health problem and its impact on quality of life. Those conclusions are based on the results of the literature review. Relationships with others were found to be important for the overall feelings of happiness of people with deafblindness, as with any other target group [3, 28]. Communication can lead to other aspects of development, especially social development and the development of self [4, 29]. Even so, people with CDB are often excluded from social contact [3, 10]. Since they have a low communicative level [2, 6], they engage in little communication with peers [3, 30] and groups [19].

Two decades ago, authors [31, 32] already stressed the importance of creating opportunities to listen to others’ communication. However, few recent publications describe MPC with people with CDB. Those that exist concern narratives [19, 33] and case descriptions [18, 34, 35]. Scientific literature about MPC between people without sensory disabilities concerns children’s development of communication skills in triadic settings [11, 36–39], development of group functioning in children [14, 40–42], and analyses of family functioning [43, 44].

Communication partners expect that having regular MPC would enhance the alertness, self-esteem, and communication level of people with CDB [19]. These expectations have been confirmed in literature about the general population. Having social relationships with others is important to a person’s general health [45–47], and MPC encourages the development of social and communicative skills [36, 41].

#### Behavior and determinants of behavior

The focus group study and the literature review provided relevant information on how the quantity and quality of MPC among people with CDB was affected by their behavior. The focus group study identified the behavioral factors of people with CDB that may affect the amount and quality of their MPC [19]. These factors are listed in the logic model (Fig 1) under “Behavior.” The determinants specific to people with CDB are listed to the left of the behavior. These determinants suggest reasons why people with CDB have limited MPC and are an important source of information for the MPC program being developed.

According to focus group participants, the dual sensory impairment makes it difficult for people with CDB to engage in incidental listening in a conversation. The focus group participants reasoned that people with residual sensory functioning may have more opportunities to overhear conversations, and they were more likely to initiate MPC with these individuals [19]. It is not yet clear how sensory functioning affects MPC. Do people with or without residual sight and/or hearing have different desires around MPC and, if so, how can this difference be explained?

Both the focus group discussions and the literature mentioned an innate need for human beings to communicate with multiple others [14, 41]. Additionally, the literature review findings indicated that the level of communication skills may influence the level of attention to other people’s conversations. In their study on infants, the authors found that 1-year-old children paid more attention to a conversation than 6-month-old children, and 3-year-old children paid even more attention [48]. In another study, authors illustrated that 3-year-old children made more predictive gaze shifts than 1-year-old children, indicating that they had better skills for following the course of a conversation [49]. Likewise, several researchers have hypothesized that growing linguistic skills support long-term triadic interactions [11, 36]. Hence, the challenges that people with CDB regularly face when developing communication skills [3] may affect their level of participation in MPC.

#### Environmental factors and determinants

Other reasons for the relatively small amount of MPC with people with CDB involve the other participants in the MPC and the environment. These are listed in the logic model (Fig 1) under “Environment.” On the left are the determinants, which help explain the reasons communication partners provide for their behavior and choices.

The report on the focus group sessions [19] offers a better understanding of the environmental factors and determinants. Some focus group participants looked to their own behavior for additional explanations. One participant suggested that professionals are unaware of the “dyadic bias” in conversations with people with CDB. Another participant indicated that MPC is not yet a habitual part of their communication strategies, and they suggested that professionals who communicate with people with CDB lack confidence in their own ability to practice MPC in the tactile modality. Training about communication strategies was part of the regular training communication partners receive, but they are not yet trained to apply these communication strategies in a multiparty setting [19].

Furthermore, several focus group participants mentioned organizational aspects that impeded MPC in their daily practice. According to focus group participants, the staffing ratio (i.e., one professional for every three to five adults with CDB) inhibits opportunities for MPC. The ratio is better (i.e., one teacher for every two to three children) at the Dutch school for children with deafblindness, but this setting also did not facilitate a natural occurrence of MPC [19].

#### Program goal

Based on the results of the needs assessment, the program goal was to increase the amount of MPC in people with CDB. Three behavioral program goals were formulated on the level of the individual with CDB. Also, four environmental program goals were formulated on the level of the intermediating group (i.e., the seeing-hearing communication partners). The behavioral program goals for the people with CDB were:

- Have more MPC
- Improve communication skills, specifically more declarative and narrative communication and new linguistic expressions
- Improve social-emotional skills, demonstrated in increased alertness, engagement, initiatives, and longer conversations

The environmental program goals for the communication partners were:

- Understand the added value of MPC and be aware of the opportunities MPC can offer to people with CDB
- Have the knowledge and skills to practice MPC with people with CDB
- Feel confident in having MPC with people with CDB
- Regularly offer MPC to the people with CDB in their network

### Step 2: Program outcomes and objectives

In the second step of intervention mapping, the working group formulated program outcomes and objectives for change by completing four tasks. The first task was to identify the stakeholders. The needs assessment identified two main target groups for the intervention: the people with CDB and their communication partners. Both groups needed to make an effort to facilitate and maintain MPC. Furthermore, since people with CDB in the Netherlands generally live in residential facilities, two more target groups were added: managers and practitioners (psychologists, coaches, speech therapists) who, respectively, facilitate MPC in daily practice and advise on MPC programs.

The second task was to identify measurable outcomes to use to create performance objectives for each stakeholder group. The sets of performance objectives for the four target groups were based on the determinants in the logic model of the problem (Fig 1). Initially, more performance objectives were formulated than were realistic for the intended duration of the program. The working group narrowed the scope by focusing on the added value of MPC rather than on objectives for communication in general, and by focusing on the changeable behavior of the target groups. The final sets of performance objectives consisted of five for the people with CDB, five for their communication partners, three for the practitioners, and two for the managers.

The third task was to identify the personal determinants that direct behavior and are changeable by interventions. Since the added value of MPC is that people learn by observing interactions between others [19], we applied observational learning from social cognitive theory [50] to explain the outcomes of the performance objectives. Bandura’s social cognitive theory describes how people learn through the experiences of others, guided by four mechanisms: attention, retention, production, and motivation [50]. Vicarious capability represents the learning that occurs by witnessing and evaluating the experiences of others. It prevents people from having to experience each situation for themselves [15]. Furthermore, the self-efficacy of the actor determines which behaviors are actually enacted. Self-efficacy refers to a sense of control over one’s own functioning in relation to the environment [51]. Both vicarious capability and self-efficacy were leading concepts in identifying the changeable determinants for the selected target groups.

When identifying and selecting determinants, we applied the intervention mapping terminology [21]. For communication partners, the selected determinants were (i) knowledge, (ii) skills, (iii) attitude/personal norms, (iv) subjective norms/social influence, and (v) self-efficacy. The determinants for people with CDB were (i) skills, (ii) self-efficacy, and (iii) habits. The determinants for practitioners were (i) knowledge, (ii) skills, (iii) attitude/personal norms, and (iv) self-efficacy. Finally, the determinants for managers were (i) knowledge, (ii) skills, (iii) attitude/personal norms, and (iv) expectations.

The final task for step 2 of intervention mapping was to link the selected determinants to the performance objectives to create matrixes of change objectives, resulting in the behavioral and environmental outcomes for the program. For example, the change objective “Communication partners express confidence in starting MPC with the person with CDB in a daily situation” was formulated with the determinant “self-efficacy” and the performance objective "Identify situations that are suitable for MPC.” Other examples of change objectives are presented in Table 2. Since both performance objectives and determinants vary between the target groups (communication partners, people with CDB, practitioners, and managers), the working group decided to construct separate matrixes for each target group. The complete matrixes are presented in S1–S4 Tables.

**Table 2:** Examples of the matrixes of change objectives for the four target groups.

Target group	Performance objective	Change objectives			
		Knowledge	Skills	Attitude/personal norms	Self-efficacy
**Communication partners**	Use accessible means of communication in the MPC	Explain which means of communication fit the sensory functioning and communication skills of the person with CDB	Use means of communication in the MPC that fit the sensory functioning and communication skills of the person with CDB	Explain the importance of using the means of communication of the person with CDB in MPC	Be creative in finding the appropriate means of communication to use with the person with CDB and the other communication partner
**People with CDB**	Show their involvement in the MPC, even when the communication is not directed at them		Display a clearly visible listening attitude during the MPC		Confidently ask for turns in the MPC
**Practitioners**	Work with communication partners to choose situations suitable for MPC with people with CDB	Recognize that an MPC does not develop naturally with people with CDB and communication partners must intentionally offer it	Describe what MPC in various situations might offer to the person(s) with CDB	Explain that MPC should be accessible to person(s) with CDB	Have confidence in one’s own abilities to support communication partners in choosing appropriate situations for MPC with the person with CDB
**Managers**	Brainstorm with communication partners to create opportunities for people with CDB to communicate with multiple communication partners simultaneously	Recognize that an MPC does not develop naturally in people with CDB and communication partners must intentionally offer it	Creatively explore opportunities to have MPC with people with CDB	Explain that it is their role to support caregivers in seeking opportunities to engage in MPC with people with CDB	

### Step 3: Program design

The third step of intervention mapping is specifying the themes, scope, and components of the program. In this step, the program itself is conceptualized based on theory- and evidence-based change methods [21].

Social cognitive theory [50] led us to several relevant change methods. The first, active learning, stimulates communication partners to set goals and practice in their own daily interactions with people with CDB. Active learning was hypothesized to influence both the skills and attitudes of communication partners. Other change methods derived from social cognitive theory include modeling (to provide relatable examples of MPC with people with CDB) and facilitation (to create optimal conditions for MPC). These two change methods aimed to increase knowledge and influence norms, respectively.

Several other change methods came from different sources. We selected the change method persuasive communication for the determinants knowledge and social norms. Persuasive communication stems from the elaboration likelihood model [52]. Finally, we chose to incorporate belief selection from the theory of planned behavior [53] with the intention to influence subjective norms. The selected theory- and evidence-based methods are listed in Table 3 and illustrated with examples of practical applications in an MPC context.

**Table 3:** Changeable determinants, theory- and evidence-based change methods, and practical applications for communication partners.

Determinant	Method	Related theory	Example of practical application
Knowledge	Modeling	SCT	Show video examples of MPC with people with CDB
	Discussion	ELM	Discuss alternative behavior
	Persuasive communication	ELM	Explain elements of conversation in MPC
	Facilitation	SCT	Identify a situation in which to start MPC
Skills/ self-efficacy	Active learning	SCT	Reflect on one’s own behavior in video-feedback coaching
Attitude/ personal norms	Persuasive communication	ELM	Explain the advantages of exemplifying behavior through MPC
	Modeling	SCT	Change attitude by reviewing examples of MPC
	Active learning	SCT	Discover through practice that it is possible to have MPC with people with CDB
Subjective norms/ social influence	Belief selection	TPB	Explain that MPC is possible for most people with CDB and how
	Modeling	SCT	Give examples of colleagues that hold MPC with people with CDB
	Persuasive communication	ELM	Give experts’ advice on how to use communication in MPC

SCT: Social Cognitive Theory; ELM: Elaboration Likelihood Model; TPB: Theory of Planned Behavior

### Step 4: Program production

This fourth intervention mapping step is dedicated to designing the program by describing its scope and structure and developing the components and materials. A basic premise for this step was that it should be a short program for communication partners. Members of the working group and managers emphasized that the time and budget available for training are limited and set by collective agreement [54]. Furthermore, existing staff shortages decrease the opportunities for training. The working group preferred to use a combination of knowledge transfer and video-feedback coaching to train communication partners because of positive experiences in earlier programs for improving the interaction and communication with people with CDB [6–8].

Based on this information and on the data collected in the previous steps, we produced a one-hour training session for communication partners. The first author drafted the session, after which the working group reviewed it. Subsequently, it was tested in a pilot training session with a team of caregivers of people with CDB, after which a few text and layout revisions were made.

The training session covered both general background information on MPC and specific aspects of it, explained in a PowerPoint presentation and illustrated with video examples of MPC with a person with CDB. The session aimed to transfer knowledge about MPC and to reflect on reasons to start using MPC in communication with the person with CDB, thereby using the change methods persuasive communication, belief selection, and discussion. The accompanying video examples provided various models for how MPC can be applied in CDB communication. Although the training session was primarily aimed at the communication partners, it also contained valuable information to help practitioners and managers achieve the program goals. Therefore, these professionals were also invited to participate in the training session.

Two of the communication partners were invited to participate in the second part of the training, consisting of alternating video-recorded MPC sessions with the person with CDB and evaluation of these sessions in video-feedback sessions with the MPC coach. Each training course had four video-feedback sessions, with a duration of 45–60 minutes each. Before each video-feedback session, the communication partners offered MPC to the person with CDB in a natural context and video-recorded the interaction. The video-feedback sessions discussed the video recording of the MPC and were guided by the communication partners’ goals and the important elements of communication in MPC. A conversation sheet was used to facilitate the sessions.

The MPC training concluded with a 45-minute evaluation with all the communication partners, the manager, and the practitioner(s). In this evaluation, the two communication partners shared their experiences with MPC, and participants made new agreements on how and when they would use MPC in the future.

The managers and practitioners were invited to join an additional general information session about MPC, the program, and their role in its application. The program did not directly target people with CDB since they were assumed to have a natural capacity to have MPC in their own language and would not need specific intervention.

### Step 5: Program implementation plan

In this fifth intervention mapping step, an implementation plan was produced. The intervention mapping approach suggests that this step be used to identify potential program users and to construct program performance and change objectives to be used in designing implementation interventions [21].

To generate a program that best reflects practice, we developed a three-stage implementation plan. In the first stage, we planned to implement the MPC program in two cases involving a person with CDB and two of their seeing-hearing communication partners. The second stage was intended to optimize use of the program. At this stage, the MPC program implementation was planned for an additional 10 cases. Results from the first two stages would be evaluated with effect studies (see step 6).

For the final phase, we planned a general implementation in residential care settings for people with sensory impairments in the Netherlands. The conditions for successful adaptation of MPC were addressed during the pilot study and the multiple-case study. Although the four care organizations had different organizational structures, the working group agreed that they all needed additional information to stimulate commitment from the indirectly involved professionals (i.e., managers and practitioners). Since these professionals were considered to be stakeholders for the program’s adoption and implementation, they should be informed early on about the added value of MPC for the care of people with CDB, the setup of the program, and the conditions for implementation (including the necessary time investment). Managers and practitioners were encouraged to identify potential study participants and discuss the study with their care professionals and legal representatives.

The working group also suggested that a program implementer (i.e., a practitioner:: psychologist, speech therapist, or care adviser) be appointed at each of the four organizations providing services for people with sensory impairments in the Netherlands. The program implementers were assigned two tasks: 1) to develop an organization-specific implementation plan that identifies and creates opportunities for implementing the MPC program in their organization; and 2) to lead MPC interventions at the client level as MPC coaches. This procedure allows the MPC program to continue to grow in the organizations. To maintain the quality of coaching and enable inter-organizational cooperation, the working group agreed that the program implementers and the principal investigator would hold bi-monthly meetings. These meetings could be a platform for evaluating the program.

### Step 6: Evaluation plan

The final intervention mapping step was to develop an evaluation plan. It included both an effect evaluation of the program and a process evaluation of its implementation. The program was intended to teach the communication partners how to offer MPC to people with CDB in order to improve the amount of MPC they engage in. Accordingly, the evaluation would need to clarify the extent to which the program achieved this goal.

The first part of the evaluation plan was an effect evaluation. Here, we selected the design for the effect study: a multiple baseline across subjects design. Since the target group is relatively small and diverse, we decided it was unrealistic to try to study a representative sample of people with CDB. A multiple baseline across subjects design has proved to be suitable for measuring effects in previous CDB research [55] and was therefore chosen for the current study. We planned to select 12 groups of three people (one person with CDB and two communication partners) to participate in the study. Inclusion criteria for the people with CDB were having CDB, being able to share attention, having no tactile defensiveness, and having no or very little experience with MPC. The effect evaluation only included MPC with one CDB partner and two seeing-hearing partners to ensure that the MPC situations were as similar as possible.

We planned to carry out data collection by analyzing video recordings of the MPC sessions and asking communication partners to complete questionnaires. Following the results of the needs assessment, we decided that video analyses would be performed in the annotation program Elan Linguistic Annotator version 6.4 [56]. To establish inter-observer reliability, two observers would individually score 10-second videos on the language expressions [57], referential communication [58], initiatives to contact [59], and attention [59] used by the people with CDB. They would also observe the use of verbal self and others in communication [60] and their symbolic communication [61]. Finally, they would assess the degree of positive affect and cohesiveness on a 5-point Likert scale [62]. A transcript of the communication would be made to assess the total number of words and the total number of unique words used in the conversations. A report of the total duration of the MPC was also planned.

Another aspect of the evaluation plan was the use of questionnaires to assess the functioning of the person with CDB (social-emotional functioning and communicative level). In addition, questionnaires would be used to assess the communication partners’ confidence and skills in practicing MPC with the person with CDB. The full methodology and outcomes of this effect study will be presented in another paper. The second part of the evaluation plan was the process evaluation, which was planned to assess the feasibility of the MPC program in practice. Data for the process evaluation would be collected through feedback from the MPC coach, the participating communication partners, the involved practitioner(s), and manager(s), and through standardized evaluation sessions with the participating communication partners. The data would be interpreted and discussed in the working group. Upon completion of the intervention studies, the program implementers and MPC coach(es) would be responsible for evaluation. Ongoing evaluations were planned to ensure that the MPC program would meet the demands of practice.

## Discussion

This article described the systematic development of a program to increase the amount of MPC with people with CDB. Although communication partners are aware that they have little MPC with people with CDB and emphasize the potential of MPC as a means to stimulate the development of communication and the social-emotional functioning of people with CDB, this realization does not automatically lead to more MPC in daily communication. Thus, the communication partners suggested that a program be developed to support them in gaining experience with MPC that involves people with CDB [19].

Intervention mapping was applied to purposefully develop such a program. This approach ensures a robust, structured, and systematic development process that involves stakeholders in the development process, is based on theory, and takes the context into account [21]. Intervention mapping has been used successfully to develop a multitude of health promotion programs (see the intervention mapping handbook for a list of examples [21]). The MPC program that emerged from this project consisted of a group training session for communication partners, four video-feedback sessions, and an evaluation. It also involved information sessions for practitioners and managers.

A strength of the intervention mapping protocol is that it is a participatory research method [63]. Following the steps ensured that the developed program was based on the experiences, needs, and solutions of the people who have daily interactions with the target group: people with CDB. The ecological approach used in intervention mapping creates interventions at different levels: individual, interpersonal, organization, and community [63]. This approach is suitable for residential care for people with intellectual disabilities, where many stakeholders are identified to support the clients [64]. Stakeholders for implementing the current study were targeted and included during the program development phase. By using this ecological approach in the development phase, we ensured that the developed program targets relevant disciplines, answers the communication partners’ questions, and is feasible in terms of time investment.

Involving existing theories on communication in the program’s development ensured that it is based on scientific evidence that children are able to participate in MPC from a very young age [12, 13] and that MPC supports the development of skills related to self-other differentiation, social coordination, sharing affect, and communication [11, 12, 14]. Theories derived from social cognitive theory about the concepts of vicarious learning and self-efficacy were used to establish and explain how MPC may enhance social and communicative development [15, 50]. People with CDB may develop these skills when they are involved more often in MPC. Since MPC does not seem to be part of a specific developmental step but is part of regular communicative environments across the lifespan [14, 40, 42], a low cognitive developmental level is not regarded as a reason to avoid introducing MPC.

Although intervention mapping has been used to develop a variety of health education programs, this was the first time it was applied to develop a program to improve communication with people with CDB, and it may have been the first time it was used in residential care for people with sensory disabilities. Intervention mapping assisted in the decision-making process during the planning, implementation, and evaluation of the MPC program. Although the application of intervention mapping is intensive and time-consuming, following the six steps of intervention mapping resulted in a rigorous program that suits the daily care and educational practice of people with CDB.

A limitation of the current study is that there is little research to be found on MPC, both in general and in relation to CDB. Thus, the program is primarily based on general theories about learning, experiences, and the practical knowledge of communication partners. We recommend that the current intervention be critically evaluated as more literature and experiences become available. Some of this will be done during the semi-annual meetings with the implementers.

Another limitation of the study is that the development of the program only involved stakeholders in Dutch specialist residential care for people with CDB. It is widely assumed that there are also many people with CDB in the Netherlands who do not receive this specialist care but instead live in intellectual disability care settings [26, 65]. The current program was aimed at people in specialist CDB care, since people with deafblindness need specific support [66], and it was hypothesized that the basic requirements for communication would be more likely to be fulfilled here than in general care. Living in a CDB specialized care setting was therefore seen as a good condition for adding MPC to the communication strategies in use. Also, the focus group sessions that addressed the lack of MPC and the potential added value for this target group consisted of communication partners of people with CDB who live in specialized care [19]. Before using the MPC program in an intellectual disability care setting, we recommend that implementers carefully examine the communication and MPC needs of those residents and the conditions for communication.

Furthermore, living situations, communication support, and possibly the nature of MPC may differ across countries, regions, and states. In the international sounding board group and in the Dutch focus group sessions, participants expressed a desire to develop an intervention to enhance MPC with people with CDB. The current article describes the development of such an intervention in the Dutch situation. But since the elements of the program, its content, and its implementation strategy must fit the general practices of the social group in question to be effective [21], people should hesitate to implement the current intervention in other countries, regions, or states. We recommend that they first study the local situation and then adapt the intervention to local practices where necessary. In addition, we recommend that results be published to generate more knowledge about MPC with people with CDB.

A final point of consideration is the broader implementation of the MPC program in the Dutch situation. Indeed, the impact of a program depends not only on its effectiveness, but also on its application in practice. Yet, implementation is extremely complicated and successful interventions do not automatically find their way into practice [67]. Some authors recommend using an active implementation strategy (i.e., a process implementation) in which experts collaborate with organizations and professionals to use the program in practice, as opposed to a passive strategy (i.e., paper implementation) that highlights the dissemination of information [68]. To facilitate process implementation in intervention mapping, other authors suggest using a strategy called implementation mapping [63]. It describes process implementation in five tasks which, like intervention mapping, are carried out iteratively [63]. Since the MPC program was developed using intervention mapping, implementation mapping may be a logical successor to optimize the implementation. Either way, if the MPC program is to be effective, a thorough implementation plan must be developed so it can find its way into the practices of communication partners of people with CDB.

## Conclusions

The intervention mapping approach led the process of developing a feasible intervention to support communication partners in having MPC with people with CDB. By involving stakeholders in the development and appointing program implementers, a foundation for a solid program has been laid. The effects of this program on the amount of MPC with people with CDB and on the development of social and communication skills of these people will be further investigated.

## Supporting information

S1 Fig Flowchart of the literature selection process for papers on the effects of MPC on social and communication skills in children. (TIF)

S1 Table Matrix of change objectives for communication partners of people with CDB. (DOCX)

S2 Table Matrix of change objectives for people with CDB. (DOCX)

S3 Table Matrix of change objectives for practitioners. (DOCX)

S4 Table Matrix of change objectives for managers. (DOCX)
