# Supplementary figures and images for: Using intervention mapping to develop an intervention for multiparty communication with people with congenital deafblindness
Source: PLoS One. 2024 May 9;19(5):e0299428. doi: 10.1371/journal.pone.0299428 (PMC11081490; doi:10.1371/journal.pone.0299428)

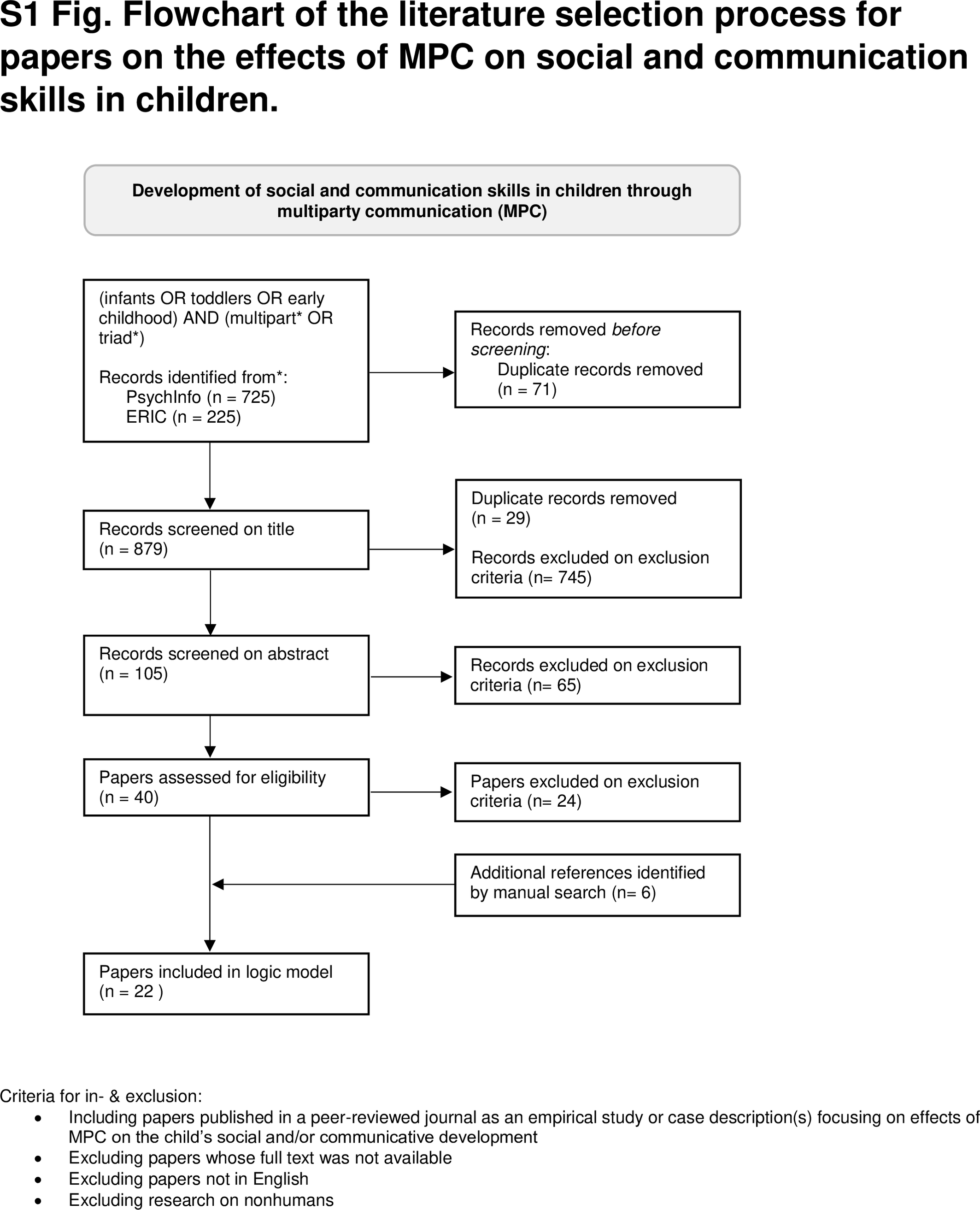

Supplement: S1 Fig — (TIF) [file pone.0299428.s001.tif]
