# Supplementary material for: Using intervention mapping to develop an intervention for multiparty communication with people with congenital deafblindness
Source: PLoS One. 2024 May 9;19(5):e0299428. doi: 10.1371/journal.pone.0299428 (PMC11081490; doi:10.1371/journal.pone.0299428)
Supplement: S1 Table — (DOCX) [file pone.0299428.s002.docx]

# S1 Table. Matrix of change objectives for communication partners of people with CDB.

**Target behavior:** Communication partners have MPC with people with CDB.

| Performance objective | Change objectives | | | | |
| --- | --- | --- | --- | --- | --- |
|  | Knowledge | Skills | Attitude/personal norms | Self-efficacy | Subjective norms/ social influence |
| Identify situations that are suitable for MPC | Label which situations may be suitable for having MPC with the individual with CDB | Find opportunities for having MPC in a daily situation of the person with CDB | Express positivity about starting MPC conversations in everyday situations with the individual with CDB | Express confidence in starting MPC with the individual with CDB | Say starting MPC with the individual with CDB is part of their job |
| Use accessible means of communication in the MPC | Explain which means of communication fit the sensory functioning and communication skills of the person with CDB | Use means of communication in MPC that fit the sensory functioning and communication skills of the person with CDB | Explain the importance of using the means of communication of the person with CDB in MPC | Be creative in finding the appropriate means of communication to use with the person with CDB and the other communication partner | Tell that they are expected to use the means of communication of the person with CDB in MPC  Tell that they are expected to keep their hands available for communication in MPC |
| Tell explicitly in accessible means of communication who enters and leaves the MPC | Appoint the steps of opening and closing the contact and explains how this is done in MPC | Explicitly introduce oneself to the other communication partner so that the person with CDB notices and understands  Leave the communicative setting following the steps of contact closure. Make sure the person with CDB notices and understands | Say it is important that the client always knows who they are communicating with | Dare to draw attention during opening and closing contact | Tell that they are expected to appropriately open and close the contact in MPC |
| Use the MPC to model aspects of social interaction and/or communication | Describe what topics and interaction elements the person with CDB could learn through modeling within MPC | Exchange information with the other communication partner within MPC, focusing on certain aspects of social interaction and/or communication. In doing so, be accessible to the person with CDB, monitoring their response | Express that modeling within MPC can help the person with CDB develop social and/or communication skills | Express confidence in modelling behavior in MPC | Say that they are expected to use modelling in MPC to teach skills to the person with CDB |
| Apply communication skills within MPC to maintain a good balance between the three communication partners | Explain which communication skills are needed for MPC with an individual with CDB | Match own communication in the MPC with both communication partners  Ensure proper turn-taking between all communication partners in MPC | Recognize that all communication partners, including the individual with CDB, have communication skills to hold their own in MPC | Express confidence in applying communication skills appropriately within MPC | Tell they are expected to apply communication skills in MPC with the person with CDB |

CDB: Congenital deafblindness

MPC: Multiparty communication
