# Supplementary material for: Using intervention mapping to develop an intervention for multiparty communication with people with congenital deafblindness
Source: PLoS One. 2024 May 9;19(5):e0299428. doi: 10.1371/journal.pone.0299428 (PMC11081490; doi:10.1371/journal.pone.0299428)
Supplement: S2 Table — (DOCX) [file pone.0299428.s003.docx]

# S2 Table. Matrix of change objectives for people with CDB.

**Target behavior:** People with CDB participate in MPC.

| Performance objective | Change objectives | | |
| --- | --- | --- | --- |
|  | Skills | Self-efficacy | Habits |
| Show their involvement in the MPC, even when the communication is not directed at them | Display a clearly visible listening attitude during the MPC  Focus attention alternately on both communication partners in MPC  Focus attention on both communication partners during listening in the MPC | Confidently ask for turns in the MPC | Maintain attention when communication is not directed at them, but at the other communication partner |
| Show that they understand that they are communicating with more than 1 person | Initiate MPC by actively seeking two communication partners or involving a second person in communication  Direct communication on both communication partners  Take a communication partner’s turn and give it to the other communication partner | Confidently involve and/or target both communication partners  Be confident in dividing roles within MPC | Not specified |
| Maintain MPC, even in case of miscommunication or non-understanding | Negotiate meaning in MPC  Be able to repair in case of miscommunication or non-understanding | Explicitly communicate vulnerability (e.g., confusion) within MPC | Make another attempt within the contact instead of breaking contact when miscommunication or non-understanding occurs |
| Introduce conversation topics in MPC | Take initiatives in the MPC, using familiar means of communication appropriate to MPC | Confidently introduce a new topic in MPC | Bring into MPC a different topic or aspect of a topic than is usual for this individual in dyadic communication |
| Respond to a topic within MPC | Address both communication partners simultaneously or switch from one to the other when reacting to a topic | Provide or request additional information within MPC | Use MPC to exchange information with multiple communication partners  Stay engaged in communication, even when it is not brief and declarative |

CDB: Congenital deafblindness

MPC: Multiparty communication
