# Supplementary material for: Using intervention mapping to develop an intervention for multiparty communication with people with congenital deafblindness
Source: PLoS One. 2024 May 9;19(5):e0299428. doi: 10.1371/journal.pone.0299428 (PMC11081490; doi:10.1371/journal.pone.0299428)
Supplement: S3 Table — (DOCX) [file pone.0299428.s004.docx]

# S3 Table. Matrix of change objectives for practitioners.

**Target behavior:** Practitioners (psychologists, coaches, speech therapists) support caregivers in having MPC with people with CDB.

| Performance objective | Change objectives | | | |
| --- | --- | --- | --- | --- |
|  | Knowledge | Skills | Attitude/personal norms | Self-efficacy |
| Work with communication partners to choose situations suitable for MPC with people with CDB | Distinguish MPC from dyadic communication and name the specific elements of MPCs  Recognize that an MPC does not develop naturally with people with CDB and communication partners must intentionally offer it  Recognize that MPC is possible at all communication levels | Explain to caregivers which situations are promising for having MPC with the individual with CDB  Describe what MPC in various situations might offer to the person(s) with CDB | Explain that MPC should be accessible to person(s) with CDB | Have confidence in one’s own abilities to support communication partners in choosing appropriate situations for MPC with the person with CDB |
| Educate communication partners in having MPC with people with CDB | Know the contents of the MPC training course materials | Not specified | Appreciate the MPC training course for improving communication partners’ skills in having MPC with people with CDB | Not specified |
| Incorporate MPC into regular treatment or support program, e.g., incorporate into day program, speech therapy, etc. | Explain how practitioners can include MPCs with people with CDB in their own work | Include MPC with people with CDB in their own work, such as in a care plan or in treatment | Not specified | Not specified |

CDB: Congenital deafblindness

MPC: Multiparty communication
