# Supplementary material for: Using intervention mapping to develop an intervention for multiparty communication with people with congenital deafblindness
Source: PLoS One. 2024 May 9;19(5):e0299428. doi: 10.1371/journal.pone.0299428 (PMC11081490; doi:10.1371/journal.pone.0299428)
Supplement: S4 Table — (DOCX) [file pone.0299428.s005.docx]

# S4 Table. Matrix of change objectives for managers.

**Target behavior:** Managers provide appropriate conditions for communication partners and people with CDB to have MPC.

| Performance objective | Change objectives | | | |
| --- | --- | --- | --- | --- |
|  | Knowledge | Skills | Attitude/ personal norms | Expectations |
| Acknowledge MPC as method and apply it in care programs | Not specified | Not specified | Emphasize the need for MPC for people with CDB in management meetings  Emphasize the need for MPC for people with CDB in team meetings of caregivers of people with CDB; stimulates support staff to have MPC | Expect the MPC training to be more accessible to support staff if it is included in the organization's care programs |
| Brainstorm with communication partners to create opportunities for people with CDB to communicate with multiple communication partners simultaneously | Recognize that an MPC does not develop naturally in people with CDB and communication partners must intentionally offer it | Creatively explore opportunities to have MPCs with people with CDB | Say that it is important for individuals with CDB to be offered MPC  Explain that it is their role to support caregivers in seeking opportunities to engage in MPC with people with CDB | Expect caregivers to more readily engage in MPC with the person with CDB if they have searched together for opportunities to do so |

CDB: Congenital deafblindness

MPC: Multiparty communication
